# Supplementary material for: The Molecular Mechanism of Body Axis Induction in Lampreys May Differ from That in Amphibians
Source: Int J Mol Sci. 2024 Feb 19;25(4):2412. doi: 10.3390/ijms25042412 (PMC10889193; doi:10.3390/ijms25042412)
Supplement: Supplementary file 1 [file ijms-25-02412-s001.zip › ijms-2848974-supplementary.pdf]

The Molecular Mechanism of Body Axis Induction in Lampreys May Differ from That in Amphibians

Galina V. Ermakova, Aleksandr V. Kucheryavyy, Andrey G. Zaraisky, Andrey V. Bayramov

SUPPLEMENTARY MATERIAL

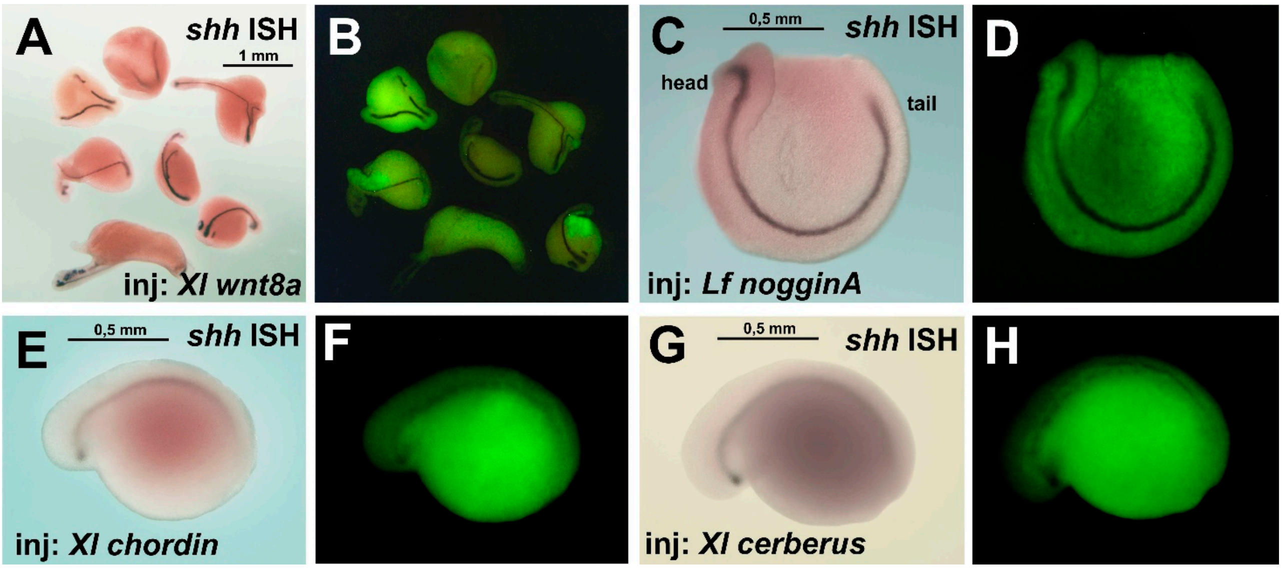

Supplementary Figure S1.
A - B – *L. fluviatilis* secondary axes diversity after injection of *Xenopus wnt8a* mRNA.
C - H – *L. fluviatilis* embryos injected with the indicated synthetic mRNAs showing no secondary axes.

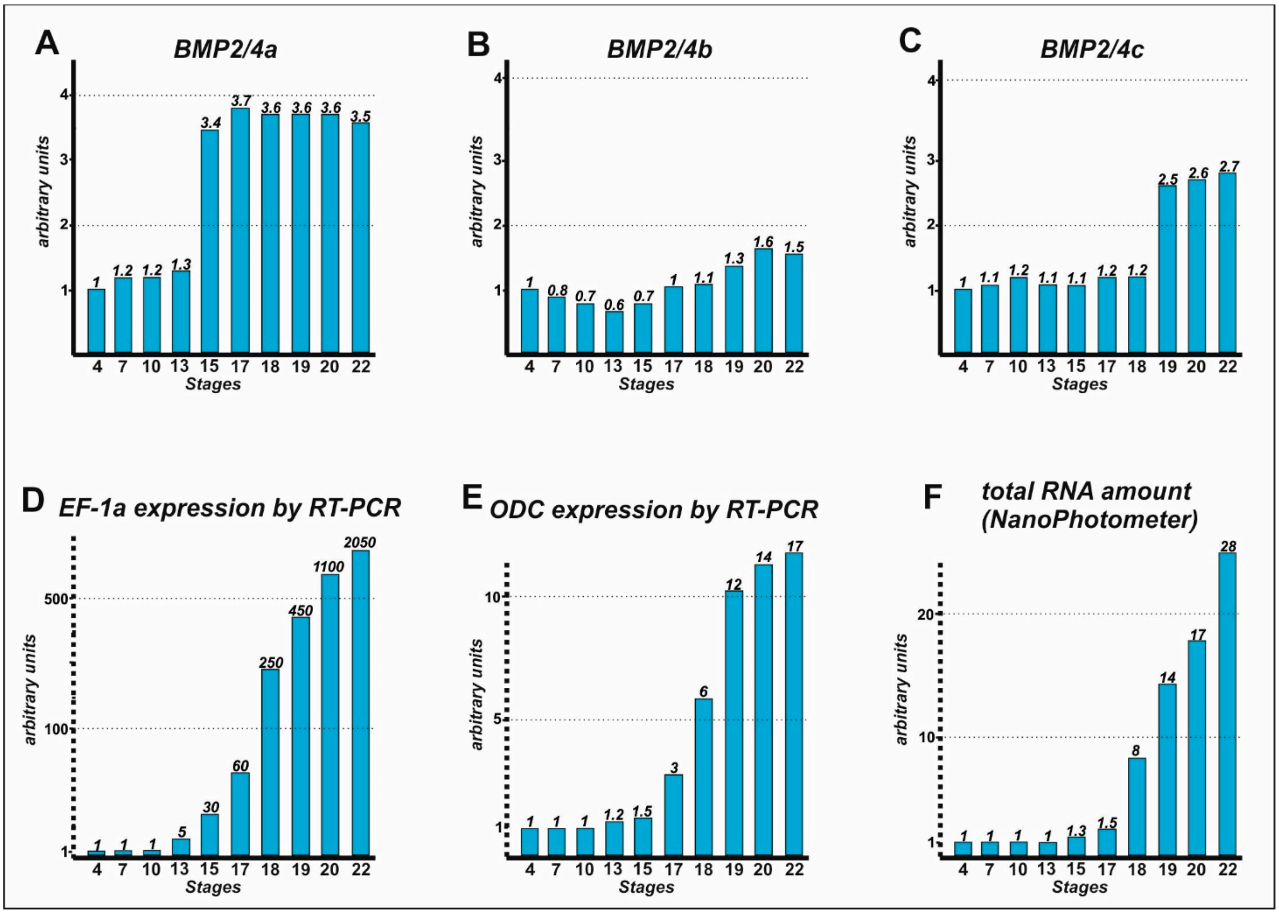

Supplementary Figure S2.

A - C - qRT-PCR analysis of *BMP2/4a*, *BMP2/4b* and *BMP2/4c* expression at early embryonic stages of *L. fluviatilis* normalized by expression of housekeeping genes *EF-1a* and *ODC*.

D - F – approximate estimate of the total amount of RNA in embryos at early embryonic stages made by qRT-PCR of housekeeping genes *ef-1a* and *odc* at a series of stages (D, E) and by measuring of the amount of total mRNA isolated from 30 embryos at each stage using Implen NanoPhotometer (F).
